# Effects of an Integrated Postural Training Program on Pain, Grip Strength, and Upper Limb Nerve Tension in Patients With Neurogenic Thoracic Outlet Syndrome: A Prospective Comparative Study

**DOI:** 10.7759/cureus.114508

**Published:** 2026-08-13

**Authors:** Akshaya V Joshi, Sandeep Shinde, Mebin S Thomas, Siddhi P Patrekar, Vaishnavi V Patil

**Affiliations:** 1 Department of Musculoskeletal Sciences, Krishna College of Physiotherapy, Krishna Vishwa Vidyapeeth (Deemed to Be University), Karad, IND; 2 Department of Geriatric Physiotherapy, Krishna College of Physiotherapy, Krishna Vishwa Vidyapeeth (Deemed to Be University), Karad, IND

**Keywords:** brachial plexus decompression, craniovertebral angle, grip strength, neurogenic thoracic outlet syndrome, posture

## Abstract

Background: Neurogenic thoracic outlet syndrome (nTOS) is a prevalent shoulder girdle disorder characterized by upper extremity pain, numbness, and motor weakness caused by mechanical compression of the brachial plexus. Faulty postural alignment, such as forward head posture and rounded shoulders, narrows the anatomical pathways, inducing neural ischemia and reduced grip strength.

Objectives: This study aimed to evaluate the effects of an eight-week Integrated Postural Training Program, compared with a Conventional Exercise Program, on pain, grip strength, upper limb nerve tension, and postural deviations in patients with nTOS.

Methodology: In this prospective comparative study, 110 participants (aged 20-50 years) with positive Adson tests and postural deviations were divided into Group A (Integrated Postural Training Program, n = 55) and Group B (Conventional Exercise Program, n = 55). Interventions lasted eight weeks (five days/week). Outcome measures included the Numerical Pain Rating Scale, hand dynamometer, occiput-to-wall distance (OWD) test, craniovertebral angle (CVA), and the brachial plexus compression test. Statistical significance was set at p < 0.0001.

Results: Postintervention, Group A demonstrated statistically significant improvements across all parameters compared with Group B (p < 0.0001). Postintervention, Group A demonstrated a significantly greater reduction in resting pain (mean difference (MD): -1.67; 95% CI: -1.94 to -1.40) and activity pain (MD: -2.74; 95% CI: -3.06 to -2.42) compared with Group B. Group A also achieved greater structural corrections, minimizing OWD substantially (MD: -2.53 cm; 95% CI: -2.78 to -2.28) and maximizing the CVA (MD: 6.26°; 95% CI: 5.20-7.32) compared with conventional exercises.

Conclusion: An eight-week Integrated Postural Training Program demonstrated greater short-term improvements in pain, grip strength, postural alignment, and upper limb nerve tension compared with a conventional exercise program in patients with nTOS. While these findings suggest that targeted postural correction is a viable conservative option, larger, blinded, randomized controlled trials with long-term follow-up are needed to confirm them.

## Introduction

The thoracic outlet is a complex, crowded structural pathway that serves as the transition zone for the brachial plexus nerve roots and major vascular channels as they exit the intervertebral foramen of the spinal cord and extend down to the axilla. This region functions as an anatomical tunnel heavily defined by boundaries including the anterior and middle scalene muscles and first rib medially, the upper trapezius muscle and scapula posteriorly, and the clavicle, coracoid process, and pectoralis minor muscle anteriorly [1]. Thoracic outlet syndrome (TOS) encompasses a complex and often controversial group of shoulder girdle disorders that arise when these critical neurovascular pathways suffer mechanical compression or irritation within these strict structural margins. It is clinically characterized by a highly variable constellation of upper extremity symptoms, including neck and arm pain, numbness, paresthesia, localized muscle weakness, and tension-related headaches [2,3].

TOS is broadly classified into two primary categories depending on the structural elements involved: vascular TOS and neurogenic TOS (nTOS). Vascular TOS is subcategorized into arterial forms (comprising approximately 1% of rare cases) and venous forms (making up roughly 3%-5% of presentations), both typically requiring aggressive clinical screening. nTOS is by far the most prevalent manifestation, accounting for over 90% of all diagnosed presentations [2]. The prevalence of TOS is significantly higher in females than in male individuals [3]. nTOS is further subdivided into two distinct clinical subtypes: "True" nTOS, a rare form marked by objective bony anomalies like a cervical rib and visible muscle wasting [4], and "Disputed" or "Non-Specific" nTOS, which represents the vast majority of clinical cases [5]. Disputed nTOS is the most common presentation overall, presenting with severe upper extremity neural symptoms without demonstrable electrophysiologic conduction abnormalities or visible muscular atrophy on standard clinical tests [4,5].

The fundamental pathophysiology underlying nTOS is rooted in compressive neuropathy, intermittent mechanical entrapment, and localized tissue ischemia. It develops when chronic biomechanical changes reduce the cross-sectional area through which the brachial plexus travels. This direct compression is associated with altered mechanosensitivity and nociceptive firing within the extraforaminal cervical nerve roots (C5-T1) and subsequent trunks of the brachial plexus. Specifically, the lower trunk (C8-T1) exhibits susceptibility to mechanical entrapment within the interscalene triangle and costoclavicular space. This compression can impair axoplasmic transport, contributing to a measurable reduction in distal hand grip strength [5].

Faulty postural alignment acts as a primary driving mechanism that initiates and exacerbates the compression seen in nTOS [6]. Modern sedentary habits and ergonomic stressors trigger a structural breakdown known as upper crossed syndrome, characterized by a forward head posture (FHP), increased thoracic kyphosis, protracted scapulae, and forward-rounded shoulders. This forward slump induces adaptive myofascial shortening and hypertrophy of the anterior/middle scalenes and pectoralis minor muscles, effectively closing the interscalene triangle and retropectoralis minor spaces. Furthermore, as postural decline causes the shoulder girdle to drop, it places continuous traction on the brachial plexus while forcing the clavicle to compress the neurovascular bundle directly against the first rib, creating a double-crush effect [6,7].

Distal motor weakness and high neural mechanosensitivity are direct functional consequences of this proximal nerve root compression. The lower trunk of the brachial plexus (C8-T1) specifically supplies the intrinsic musculature of the hand; when it is subjected to chronic costoclavicular traction and compression, axoplasmic transport is impaired. This leaves patients with a noticeable reduction in hand grip strength, which severely diminishes their functional upper extremity coordination during daily tasks. Concurrently, this physical distortion imposes elevated neural tension across the entire median, radial, and ulnar nerve tracts, causing hyperirritability that can be easily provoked during neurodynamic clinical assessments like the brachial plexus compression test [5,6].

Surgical options for TOS focus on expanding constricted neurovascular pathways through invasive techniques like first rib resection, anterior/middle scalenectomy, or pectoralis minor tenotomy. While these transaxillary, supraclavicular, or infraclavicular procedures provide substantial functional relief for neurogenic cases and high success rates for vascular conditions, their close proximity to vital thoracic and cervical anatomy introduces serious risks. Potential complications include long thoracic or phrenic nerve injuries, pneumothorax from pleural breaches, localized vascular damage, and symptom recurrence driven by dense postsurgical scarring [8]. Consequently, these invasive operations remain secondary interventions reserved for refractory cases.

In contrast, comprehensive conservative management offers a safer, efficacious first-line strategy that systematically alleviates peripheral nerve mechanosensitivity and enhances hand coordination, successfully preventing symptom recurrence and optimizing long-term quality of life without structural risks. While conservative management is considered first-line, standardized protocols remain lacking, justifying further comparative studies.

This study aims to establish an evidence-based physiotherapy protocol for nTOS by evaluating an integrated postural training program against a conventional exercise program. Designed to address the mechanical root causes of brachial plexus compression, the patient-centered program targets upper quadrant deviations common in sedentary lifestyles. These include FHP, thoracic kyphosis, protracted scapulae, and rounded shoulders, all of which cause the adaptive muscle shortening that narrows the interscalene and costoclavicular spaces. By utilizing a robust methodology with validated, objective outcome measures, this study will assess and compare the distinct impacts of the interventions on pain, grip strength, upper limb nerve tension, and postural deformities. Consequently, this research seeks to fill existing literature gaps, providing clinicians with a standardized framework to prevent symptom recurrence, restore upper limb function, and optimize long-term quality of life for nTOS patients.

## Materials and methods

This was a comparative study conducted at Krishna Vishwa Vidyapeeth (Deemed to be University), Karad, from November 13, 2025, to May 31, 2026. The minimum sample size was calculated using the formula $n = \frac{Z^2 p q}{L^2}$, where n represents the required sample size, the Z value was set to 1.96, which corresponds to a 95% confidence interval (CI); p represented the estimated prevalence of nTOS (12%) [9]; q was calculated as 1 - p (88%); and L denoted the allowable margin of error (5.86%). ​​​Based on these parameters, the initial sample size requirement was determined to be 118 participants.

This study included 118 participants, including male and female participants between 20 and 50 years of age who have tested positive for the Adson test indicating nTOS and who exhibit at least one postural deviation, such as FHP, increased thoracic kyphosis, or rounded shoulders [2]. Conversely, individuals were excluded if they had vascular types of TOS (venous or arterial), pregnancy, postoperative stiffness conditions associated with postural deviations, or a positive Spurling test indicating cervical spondylosis [10,11]. Additionally, candidates presenting with any medical red flags (including cauda equina syndrome, malignancy or tumors, spinal infections, or spinal fractures) or psychological yellow flags (such as maladaptive beliefs like catastrophizing, fear-avoidant behaviors that cause physical deconditioning, or emotional distress involving anxiety, depression, and helplessness regarding their physical condition) will be excluded from participation.

A total of 118 individuals were initially evaluated for eligibility to meet the expected sample size requirement. Of these, eight individuals were excluded from the final study: five participants did not meet the specific inclusion parameters, and three participants declined to provide consent. This resulted in a final sample size of 110 participants. Notably, no dropouts were reported during the eight-week intervention period following the initial allocation.

**Figure 1:**
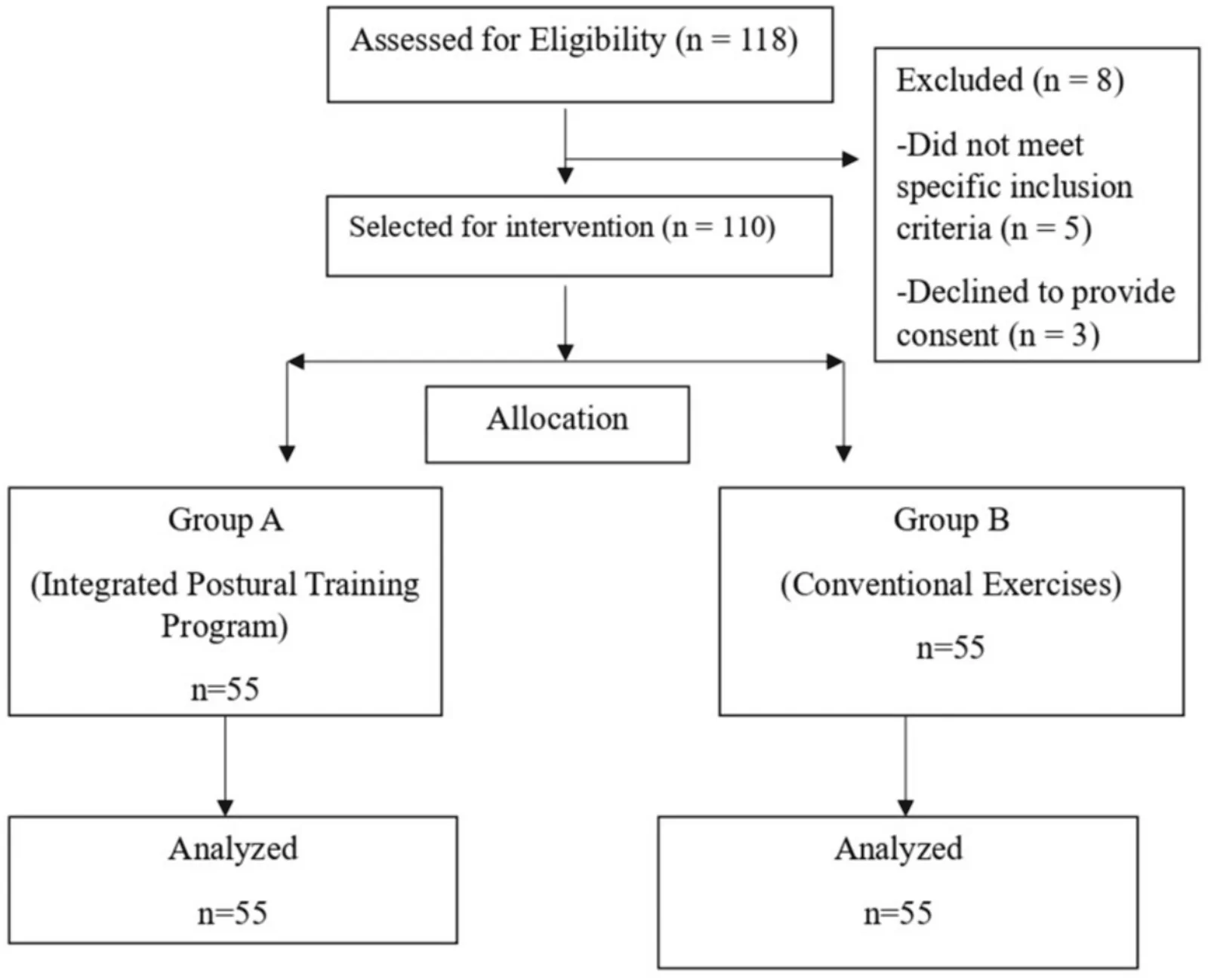
Study flowchart

Participants were allocated to one of the two intervention groups using a simple randomization sequence generated by an independent researcher not involved in the trial. To ensure robust allocation concealment, the assignments were secured using sequentially numbered, opaque, sealed envelopes. Envelopes were opened sequentially by the trial coordinator only after the participant had completed baseline testing and met all eligibility criteria. Due to the active nature of the physical interventions and local institutional constraints, blinding of the participants, treating therapists, and outcome assessors was not possible. Consequently, this trial is classified as an open-label comparative study with an acknowledged high risk of performance and detection bias. To minimize measurement bias within these constraints, all clinical endpoints were evaluated using highly structured, objective, and standardized assessment tools. Figure 1 provides a study flowchart.

Prior to its initiation, this study obtained ethical clearance from the Institutional Ethics Committee of Krishna Vishwa Vidyapeeth (Deemed to be University), Karad (Protocol Number: 277/2025-2026). The research was conducted in strict compliance with the ethical standards established by the Declaration of Helsinki. Before participating, all subjects provided informed written consent, following explicit assurances regarding complete data privacy and their right to withdraw from the study at any time without penalty or negative consequences.

Outcome measures

Numerical Pain Rating Scale

The Numerical Pain Rating Scale (NPRS) was utilized to evaluate pain intensity. Participants selected an integer from an 11-point scale (0-10) to indicate the severity of pain [11,12].

Hand Dynamometer

Grip strength was evaluated using a calibrated handheld dynamometer. Participants were instructed to sit comfortably with their shoulder adducted, elbow flexed at 90°, and forearm in a neutral position. Upon a verbal cue, participants were asked to squeeze the handle with maximum isometric effort for three seconds. The device recorded the peak force exerted. To ensure data reliability, three consecutive trials were performed with a 60-second rest interval between attempts, and the mean (or maximum) value was recorded for analysis [13].

Occiput-to-Wall Test

Postural alignment and thoracic spine kyphosis were evaluated using the occiput-to-wall distance (OWD) test, a validated clinical screening tool. Participants were instructed to stand barefoot with their heels, buttocks, and scapulae placed firmly against a flat, vertical wall while maintaining an upright posture and looking straight ahead (ensuring the Frankfurt plane was horizontal). The examiner measured the horizontal distance from the most prominent part of the occiput to the wall using a standard tape measure or rigid ruler aligned perpendicular to the wall. Measurements were recorded to the nearest 0.1 cm. An OWD value greater than 5 cm was established as the clinical threshold indicating abnormal thoracic kyphosis [14].

Craniovertebral Angle

The craniovertebral angle (CVA) is a highly reliable objective measure used to quantify the severity of FHP through sagittal-view clinical photography or computerized postural analysis. It is calculated by identifying the intersection of two lines: a horizontal line drawn directly through the spinous process of the seventh cervical vertebra (C7), and a line connecting the C7 spinous process to the tragus of the ear. Because the angle is measured relative to this horizontal plane, the CVA is inversely proportional to the degree of forward head protrusion. A normal, well-aligned posture typically yields a CVA of >48° to 50°, whereas a smaller angle indicates significant FHP [15].

Brachial Plexus Compression Test

To perform the brachial plexus compression test, the examiner applies firm pressure to the brachial plexus over the trunks of the brachial plexus within the supraclavicular fossa (specifically just posterior to the sternocleidomastoid muscle and anterior to the trapezius). Localized pain at the compression site is not diagnostic; the test is considered positive only if the pain radiates into the shoulder or upper extremity. A positive result indicates the presence of a cervical spine lesion with an underlying mechanical component [11,16].

A total of 110 participants were equally allocated into two groups of 55: Group A received an Integrated postural training program, whereas Group B underwent conventional exercises. Both groups received hot fomentation and interferential therapy as baseline treatments. The intervention spanned eight weeks, with exercise sessions administered five times a week to maintain consistent physiological loading. Exercise difficulty progressed from weeks 1 to 8 based on individual tolerance and performance. Additionally, physical training was supplemented with patient education focusing on postural awareness, workplace ergonomics, and mindfulness practices. Table 1 outlines the eight-week treatment protocols for both Groups A and B.

**Table 1:** Eight-week integrated postural training program ROM: range of motion; NPRS: Numerical Pain Rating Scale

Week	Exercise type and explanation	Dosage (sets × reps/hold)	Progression criteria
Weeks 1-2	Breathing exercises: inhale through your mouth, hold the breath for 30 seconds, and then exhale through your mouth	20-30 seconds hold × 3-5 times	Baseline entry criteria (positive Adson test, forward head posture/rounded shoulders). Exercises performed in gravity-eliminated or supported positions (standing/sitting)
	Stretching: Pectoral stretch - to perform a doorframe pectoral stretch, stand in an open doorway with your forearms flat against the frame on either side, elbows bent at a 90° angle at shoulder height. Step one foot forward to stabilize your balance and slowly lean your body weight forward until you feel a comfortable stretch across your chest without shrugging your shoulders. Hold this position while breathing normally. Trapezius stretch: to perform an upper trapezius stretch, and sit or stand tall with good posture and lower your right shoulder down toward the floor. Gently tilt your left ear toward your left shoulder, and for a deeper stretch, place your left hand lightly on top of your head to guide it further, ensuring you do not pull or force the movement. Hold this position while breathing deeply. Levator scapulae stretch: sit or stand tall and drop your right chin down toward your chest, then rotate your head about 45° to the left, as if looking down into your left coat pocket. To gently deepen the stretch, place your left hand on the back of your head to apply a light downward guide while keeping your right shoulder pressed down; you can actively anchor it by holding onto the bottom of your chair. Hold this position while breathing normally	20-30 seconds hold × 3-5 times	
	Cervical stabilization exercises on an unstable surface: sitting on a gym ball, perform all ROM exercises of the cervical, thoracic, and lumbar spine. Quadrupod position should be performed over a gym ball: ask the patient to perform cervical flexion and extension, and thoracic spine flexion and extension. Pressing the ball against the wall: ask the patient to place the ball between the wall and their head, then press it and hold. Advance multiplanar stabilization exercises: ask the patient to press the ball against the wall while holding the resistance band in both hands, and retraction should be done	10 seconds hold × 10 times	
	Wall angle exercises in standing position: U position - patient should be in standing with shoulder and elbow 90° abducted, make a “U” shape, and retract the shoulder blades close together and hold; W position - patient should be in standing with shoulder and elbow 90° abducted, make a “W” shape, and retract the shoulder blades close together and hold; T position - patient should be standing with the shoulder abducted to 90° and elbow extended, make a “T” shape, and retract the shoulder blade close together and hold; Y position - patient should be in standing with the shoulder abducted to 120° and elbow extended, make a “Y” shape, and retract the shoulder blade close together and hold	10 seconds hold × 10 times	
	Nerve gliding exercises - for median nerve (tension technique) - elbow extended, wrist extended, contralateral lateral flexion of the neck (sliding technique); Proximal - wrist flexion, elbow extension, contralateral lateral flexion of neck; Distal - wrist extension, elbow extension, ipsilateral lateral flexion of neck; For ulnar nerve (tension technique) - elbow flexion, wrist extension, forearm pronated shoulder abduction to 90° contralateral flexion of the neck (sliding technique); Proximal - decrease elbow flexion and wrist extension, and subsequently move the head towards the hand; Distal - increase elbow extension and wrist extension; For radial nerve (tension technique) - shoulder and forearm into internal rotation and pronation, and contralateral lateral neck flexion (sliding technique); Flexing the wrist with simultaneous movement of the head toward the opposite side	10 seconds hold × 10 times	
Weeks 3-4	Scapular retraction exercises in prone lying: U position - patient should be in prone lying with shoulder and elbow 90° abducted, make “U” shape, and retract the shoulder blades close together and hold; W position - patient should be in prone lying with shoulder and elbow 90° abducted, make a “W” shape, and retract the shoulder blades close together and hold; T position - patient should be in prone lying with the shoulder abducted to 90° and elbow extended, make “T” shape, and retract the shoulder blade close together and hold; Y position - patient should be in prone lying with the shoulder abducted to 120° and elbow extended, make “Y” shape, and retract shoulder blades close together and hold	10 seconds hold × 10 times	Progression permitted if the patient achieves a ≥2-point reduction on the NPRS during active movement, shows zero peripheralization of symptoms during nerve glides, and demonstrates correct form during supported wall angles
	Thoracic spine mobility drill - wall rotations with the TheraBand. The patient should be in a half-kneeling position, hold their hand in front of their body, take wall support, and rotate the other hand horizontally. Foam roller extension - lie down supine, place the foam roller horizontally under the thoracic region. Bend both knees, place both hands under the head, and move the body to and fro using a foam roller. Side-lying rotation - the patient should be in a side-lying position with one side hip and knee flexed 90° over the other leg, and rotation of one arm should be done	15 repetitions for the first 7 days; for the first 7 days, perform 15 repetitions. For the remaining 7 days, perform 10-second holds × 10 repetitions using a TheraBand as a progression	
	Grip strengthening exercises: Hand grippers - use a spring-loaded torsion gripper. Focus on full closures where the handles touch. Dumbbell forearm wrist curls - sit on a bench, resting your forearm on your thigh with your palm facing up, holding a dumbbell. Let the weight roll down to your fingertips, then curl the dumbbell back up into your palm by flexing your wrist	10 seconds hold × 10 times	
Weeks 5-8	Shoulder blade squeeze exercises using swiss ball (ask the patient to perform the quadripod position): U position - patient should be in prone lying over the swiss ball with shoulder and elbow 90° abducted, make “U” shape, and retract shoulder blades close together and hold; W position - patient should be in prone lying over the swiss ball with shoulder and elbow 90° abducted, make a “W” shape, and retract shoulder blades close together and hold; T position - patient should be in prone lying over the swiss ball with the shoulder abducted to 90° and elbow extended, make “T” shape, and retract shoulder blades close together and hold; Y position - patient should be in prone lying over the swiss ball with the shoulder abducted to 120° and elbow extended, make “Y” shape, and retract shoulder blades close together and hold	10 seconds hold × 10 times	Progression permitted if the patient successfully handles TheraBand (Performance Health, Akron, OH) resistance without pain spikes (NPRS ≤3 during activity) and displays improved thoracic extension on the foam roller
	Scapular control exercises - Inferior glide - isometric exercise focuses on humeral depression and shoulder retraction. Patient sits upright with the side to be trained in 90° abduction and fist clench, and ask the patient to apply pressure and inferiorly depress the scapula; Low row - scapular external rotation and posterior tilt; patient stands in front of the treatment bench with the hand of the side to be trained placed on the edge of the bench and palm facing posteriorly, then the patient extends his trunk and maximally pushes his hand against the edge of the bed. Lawn Mower exercise with Thera Belt - it is a multijoint exercise. Trunk extension, trunk rotation, and scapular retraction. Patient starts with his trunk flexed and rotated to the contralateral side to be exercised so that his hand is at knee height and is asked to rotate and extend trunk toward the side of training and place his elbow into his back pocket	10 seconds hold × 10 times	

To ensure maximum safety, diagnostic precision, and strict adherence to the biomechanical protocols, all exercise sessions for Groups A and B were conducted under 100% direct supervision. Every session was administered on a one-on-one basis by a physical therapist. The supervision enabled precise monitoring of postural alignment and immediate adjustment of exercise parameters if symptom exacerbation occurred during movement.

**Figure 2:**
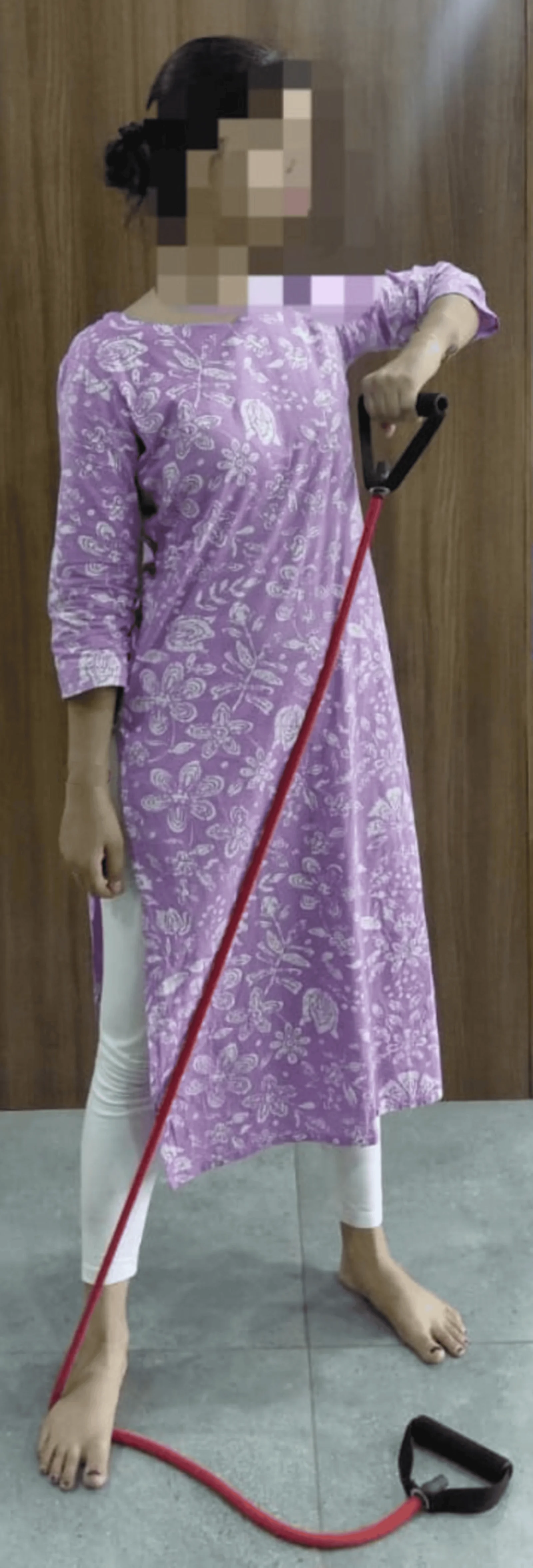
Individual performing integrated postural training exercise

**Figure 3:**
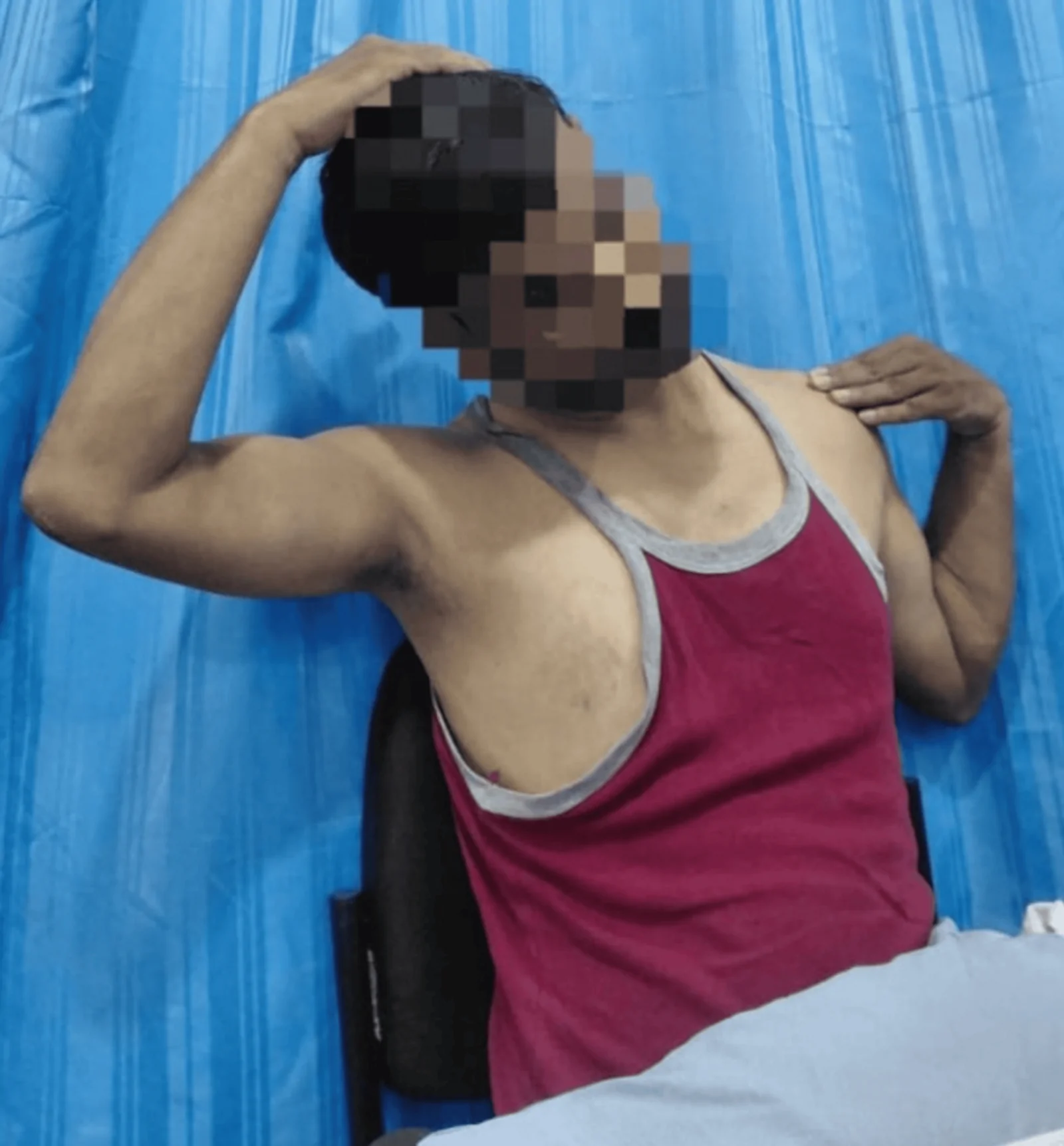
Individual performing conventional exercise

For Group B, the prescribed regimen is a comprehensive program targeting breathing, flexibility, stabilization, and mobility. It begins with deep breathing techniques and targeted stretches for the pectorals, upper trapezius, and levator scapulae, which are held for 20-30 seconds and repeated three to five times. To build strength and stability, the routine includes cervical stabilization movements like chin tucks and isometrics, alongside scapular retraction exercises such as wall angles (U, W, T, Y) and ball presses, all held for 10 seconds across 10 repetitions. Finally, the program is rounded out with dynamic movements consisting of 10 repetitions each for thoracic wall rotations and general range-of-motion exercises for the cervical, thoracic, and shoulder joints. The implementation of the integrated postural training program is demonstrated in Figure 2, and the conventional exercise protocols are demonstrated in Figure 3.

Statistical analysis

Data were processed using IBM Statistical Package for the Social Sciences Statistics for Windows, version 26 (Released 2018; IBM Corp., Armonk, NY) and validated through manual analysis. Within-group pre- and postintervention changes were analyzed using paired t-tests, whereas independent t-tests were utilized for all posttest between-group comparisons. To provide a measure of clinical magnitude and precision, mean differences (MDs) with corresponding 95% CIs were calculated for all outcome measures. The primary analysis was conducted following intention-to-treat principles; however, because there were no participant dropouts or missing data throughout the eight-week intervention, data imputation techniques were not required. To ensure the reproducibility of clinical outcomes and minimize type I error inflation, a conservative alpha level of p < 0.0001 was established as the criterion for statistical significance.

## Results

Table 2 summarizes the baseline demographics of the participants.

**Table 2:** Demographic of the study participants

Variable	Group A	Group B
Age (years)		
20-30	22	18
31- 40	13	29
41-50	20	8
Gender		
Male	21	28
Female	34	27

Table 3 illustrates a comparison of pre- and posttest mean scores of NPRS within Groups A and B.

**Table 3:** Comparison of pre- and posttest mean scores of Numerical Pain Rating Scale within Groups A and B A paired t-test was used to calculate within-group analysis and p values Statistical significance is set at p < 0.0001 SD: standard deviation; CI: confidence interval

Groups	Pretest (mean ± SD)	Posttest (mean ± SD)	Within-group mean difference (95% CI)	t value	p value
At rest					
Group A	5.24 ± 0.87	1.45 ± 0.63	-3.79 (-4.08 to -3.50)	26.34	<0.0001
Group B	5.31 ± 0.91	3.12 ± 0.78	-2.19 (-2.50 to -1.88)	14.12	<0.0001
On activity					
Group A	7.85 ± 1.12	2.11 ± 0.74	-5.74 (-6.10 to -5.38)	32.18	<0.0001
Group B	7.91 ± 1.08	4.85 ± 0.95	-3.06 (-3.43 to -2.69)	16.45	<0.0001

Both groups demonstrated statistically significant within-group reductions in pain intensity from baseline to the eight-week posttest for both resting and activity conditions (p < 0.0001). Within Group A, resting pain decreased by a mean of 3.79 points (95% CI: -4.08 to -3.50) and activity pain decreased by 5.74 points (95% CI: -6.10 to -5.38). Within Group B, resting pain decreased by a mean of 2.19 points (95% CI: -2.50 to -1.88) and activity pain decreased by 3.06 points (95% CI: -3.43 to -2.69). Table 4 illustrates comparison of pre- and posttest mean scores of hand dynamometer within Groups A and B.

**Table 4:** Comparison of pre- and posttest mean scores of hand dynamometer within Groups A and B A paired t-test was used to calculate within-group analysis and p values Statistical significance is set at p < 0.0001 SD: standard deviation; CI: confidence interval

Groups	Pretest (mean ± SD)	Posttest (mean ± SD)	Within-group mean difference (95% CI)	t value	p value
Dominant side					
Group A	32.45 ± 4.12	41.85 ± 3.88	9.40 (8.38 to 10.42)	-18.42	<0.0001
Group B	32.18 ± 4.05	35.62 ± 3.92	3.44 (2.32 to 4.56)	-6.14	<0.0001
Nondominant side					
Group A	28.12 ± 3.75	35.94 ± 3.42	7.82 (6.79 to 8.85)	-15.18	<0.0001
Group B	28.40 ± 3.68	30.85 ± 3.55	2.45 (1.53 to 3.37)	-5.32	<0.0001

Both groups achieved statistically significant within-group gains in bilateral hand grip strength from baseline to postintervention (p < 0.0001). Within Group A, dominant side grip strength increased by a mean of 9.40 kg (95% CI: 8.38-10.42) and nondominant grip strength increased by 7.82 kg (95% CI: 6.79-8.85). Within Group B, dominant side grip strength increased by a mean of 3.44 kg (95% CI: 2.32-4.56) and nondominant grip strength increased by 2.45 kg (95% CI: 1.53-3.37). Table 5 illustrates comparison of pre- and posttest mean scores of the occiput-to-wall test within Groups A and B.

**Table 5:** Comparison of pre- and posttest mean scores of the occiput to wall test within Groups A and B A paired t-test was used to calculate within-group analysis and p values Statistical significance is set at p < 0.0001 SD: standard deviation; CI: confidence interval

Groups	Pretest (mean ± SD)	Posttest (mean ± SD)	Within-group mean difference (95% CI)	t value	p value
Group A	5.42 ± 1.15	1.12 ± 0.45	-4.30 (-4.65 to -3.95)	24.85	<0.0001
Group B	5.38 ± 1.20	3.65 ± 0.82	-1.73 (-2.04 to -1.42)	11.24	<0.0001

Both cohorts demonstrated statistically significant within-group reductions in OWD following their respective eight-week interventions (p < 0.0001). Group A achieved a mean reduction of 4.30 cm (95% CI: -4.65 to -3.95), bringing postintervention measurements down to 1.12 ± 0.45 cm. Group B achieved a mean reduction of 1.73 cm (95% CI: -2.04 to -1.42), leaving a postintervention measurement of 3.65 ± 0.82 cm. Table 6 compares pre- and posttest mean scores of the CVA within Groups A and B.

**Table 6:** Comparison of pre- and posttest mean scores of the craniovertebral angle within Groups A and B A paired t-test was used to calculate within-group analysis and p values Statistical significance is set at p < 0.0001 SD: standard deviation; CI: confidence interval

Groups	Pretest (mean ± SD)	Posttest (mean ± SD)	Within-group mean difference (95% CI)	t value	p value
Group A	38.45 ± 3.12	47.82 ± 2.65	9.37 (8.41 to 10.33)	-19.54	<0.0001
Group B	38.18 ± 3.25	41.56 ± 2.94	3.38 (2.39 to 4.37)	-6.85	<0.0001

**Figure 4:**
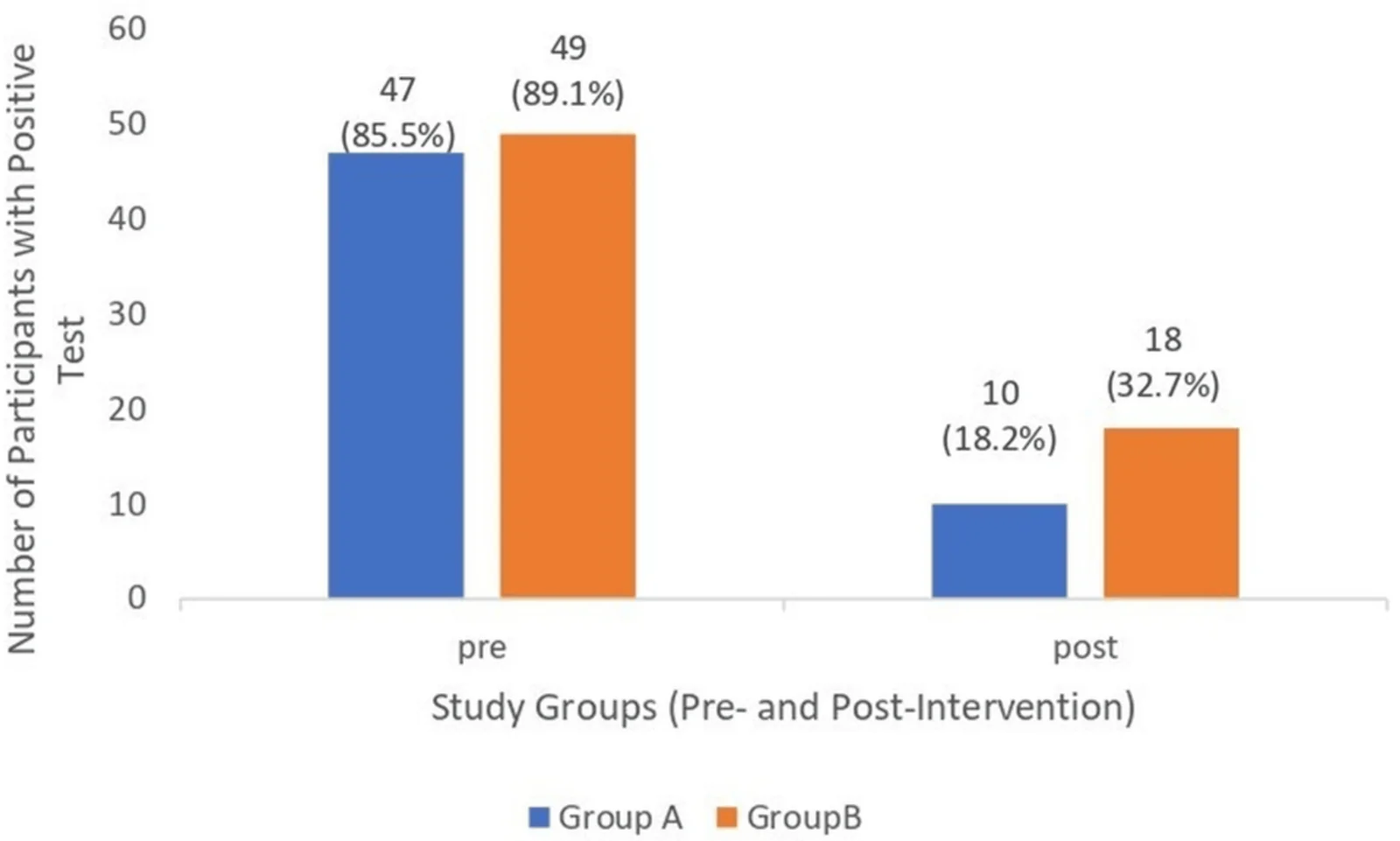
Comparative distribution of pre- and postintervention positive brachial plexus compression test between Groups A and B The Y-axis represents the participant count and percentage within each group who tested positive for the brachial plexus compression test, whereas the X-axis displays the preintervention (baseline) and postintervention (eight weeks) status for the cohorts. Group A (integrated postural training program) demonstrated a significantly higher recovery rate compared to Group B (conventional exercise program) (p < 0.0001)

Both interventions yielded statistically significant within-group increases in the CVA from baseline (p < 0.0001). Within Group A, the CVA increased by a mean of 9.37° (95% CI: 8.41-10.33), reaching 47.82° ± 2.65° postintervention. Within Group B, the CVA increased by a mean of 3.38° (95% CI: 2.39-4.37), reaching 41.56° ± 2.94° postintervention. Figure 4 illustrates a comparison of the pre- and postintervention positive brachial plexus compression test between Groups A and B.

The provided bar chart illustrates the comparative frequency of positive brachial plexus compression test results between the two groups before and after their respective interventions. At baseline, both cohorts exhibited highly homogeneous neural mechanosensitivity profiles, with a significant majority of participants testing positive for brachial plexus compression, comprising 47 out of 55 individuals (85.5%) in Group A and 49 out of 55 individuals (89.1%) in Group B. Following the intervention period, both groups achieved highly statistically significant within-group reductions in neural compression frequencies. However, Group A demonstrated a statistically higher success rate, successfully rendering 37 patients negative on the compression test, leaving only 10 individuals (18.2%) still testing positive postintervention. In contrast, Group B showed a more limited therapeutic clearance, with 18 individuals (32.7%) remaining positive for neural tissue mechanosensitivity. These findings indicate that while standard exercise successfully mobilizes neural structures, the integrated postural protocol offers a significantly more robust resolution of brachial plexus dural compression, likely by optimizing cervicothoracic alignment and reducing the duration or severity of mechanical entrapment or adverse neural tissue elongation. This angular restoration was verified by a significant between-group posttest MD of 6.26° (95% CI: 5.20-7.32) in favor of Group A. Table 7 illustrates comparison of posttest mean scores of the outcome measures between Groups A and B.

**Table 7:** Comparison of posttest mean scores of the outcome measures between Groups A and B Statistical significance is set at p < 0.0001 SD: standard deviation; CI: confidence interval

Outcome measures	Group A (mean ± SD)	Group B (mean ± SD)	Within-group mean difference (95% CI)	t value	p value
NPRS					
At rest	1.45 ± 0.63	3.12 ± 0.78	-1.67 (-1.94 to -1.40)	-12.35	<0.0001
On activity	2.11 ± 0.74	4.85 ± 0.95	-2.74 (-3.06 to -2.42)	-16.87	<0.0001
Hand dynamometer					
Dominant	41.85 ± 3.88	35.62 ± 3.92	6.23 (4.76 to 7.70)	8.38	<0.0001
Nondominant	35.94 ± 3.42	30.85 ± 3.55	5.09 (3.77 to 6.41)	7.66	<0.0001
Occiput to wall test	1.12 ± 0.45	3.65 ± 0.82	-2.53 (-2.78 to -2.28)	-20.06	<0.0001
Craniovertebral angle	47.82 ± 2.65	41.56 ± 2.94	6.26 (5.20 to 7.32)	11.73	<0.0001

Independent t-tests assessing postintervention outcomes between the two cohorts confirm statistically significant differences favoring Group A across all outcome measures (p < 0.0001). Posttest comparisons demonstrate that Group A achieved significantly lower resting pain (MD: -1.67; 95% CI: -1.94 to -1.40) and activity pain (MD: -2.74; 95% CI: -3.06 to -2.42) compared with Group B. Additionally, Group A demonstrated superior postintervention grip strength on both the dominant (MD: 6.23 kg; 95% CI: 4.76-7.70) and nondominant sides (MD: 5.09 kg; 95% CI: 3.77-6.41), as well as greater structural corrections in OWD (MD: -2.53 cm; 95% CI: -2.78 to -2.28) and CVA (MD: 6.26°; 95% CI: 5.20-7.32).

## Discussion

The primary objective of this study was to evaluate the therapeutic efficacy of an eight-week integrated postural training program (Group A) compared with a conventional exercise program (Group B) in patients diagnosed with nTOS. The results demonstrated that while both interventions yielded statistically significant improvements from baseline (p < 0.0001), the integrated postural protocol achieved distinct clinical and statistical superiority across all evaluated outcome measures. At baseline, both patient cohorts exhibited homogeneous clinical profiles with notably severe impairments. This was heavily characterized by advanced FHP, diminished bilateral grip strength, and highly prevalent brachial plexus tissue mechanosensitivity.

The structural foundation of these symptoms is deeply rooted in the biomechanical collapses associated with Upper Crossed Syndrome. As sedentary ergonomics force a forward slump, adaptive shortening and muscle hypertrophy of the scalenes and pectoralis minor directly compromise the interscalene triangle and retropectoralis minor spaces. This spatial reduction imposes physical compression and traction on the brachial plexus nerve roots, prompting localized perineural edema, ischemia, and heightened nociceptive firing [7]. This baseline severity was clearly reflected in our cohort's high initial NPRS scores, which were predictably exacerbated during active physical movement.

The study by Pascarelli and Hsu evaluated 485 patients comprising 70% computer users, 28% musicians, and 2% other repetitive workers experiencing work-related upper extremity pain and weakness. Through comprehensive physical assessments, researchers discovered that while symptoms often presented distally in the hands and wrists, the underlying condition was actually a diffuse neuromuscular illness heavily rooted in proximal upper body issues. Key clinical findings included high rates of postural misalignment, such as protracted shoulders (78%) and forward head position (71%), alongside prominent conditions like cubital tunnel syndrome (64%), medial epicondylitis (60%), and posture-related nTOS (70%). The authors concluded that nTOS acts as a primary catalyst in a cascading series of physical events, causing these disorders. In conclusion, they emphasized that comprehensive upper body clinical examinations are vital, as they uncover complex neuromuscular dysfunction that standard laboratory tests and surveys frequently miss [17].

Luu et al. conducted a scoping review evaluating conservative exercise protocols for nTOS. Their findings highlight that targeted exercise rehabilitation significantly reduces pain and improves functional outcomes. The review emphasizes a multimodal approach combining stretching of hyperactive anterior musculature, specifically the scalenes and pectoralis minor, with strengthening of the deep cervical flexors and scapular stabilizers. Maximizing costoclavicular decompression and incorporating neural mobilization are identified as critical components for successful nonoperative management and preventing symptom recurrence [18].

Balderman et al. evaluated physical therapy (PT) and surgical outcomes in a prospective cohort of 563 nTOS patients. The study highlighted that a structured, specialized PT program focusing on stretching, strengthening, and postural education successfully resolved symptoms in over 30% of patients, preventing surgical intervention. For those requiring supraclavicular decompression and scalenectomy, surgery provided substantial, long-term improvement in patient-reported outcome measures, demonstrating that conservative management is an effective first-line therapy, with surgical decompression remaining a highly successful secondary option [19].

The integrated postural training program demonstrated greater short-term clinical improvements over conventional protocols by targeting the global upper quarter kinetic chain rather than localized symptoms. By addressing components of upper crossed syndrome, stretching hyperactive pectoralis minor and scalene structures while strengthening underactive deep cervical flexors and scapular stabilizers, the program achieved significant structural realignments and functional gains within the study cohort.

This therapeutic impact was supported across multiple primary outcomes. For pain intensity, Group A achieved an additional reduction in activity pain of 2.74 points on the NPRS (95% CI: -3.06 to -2.42) compared with Group B, exceeding the established minimal clinically important difference for mechanical upper quadrant pain. In terms of distal neuromuscular function, Group A demonstrated notable gains in hand grip strength, exhibiting a postintervention between-group MD of 6.23 kg (95% CI: 4.76-7.70) on the dominant side and 5.09 kg (95% CI: 3.77-6.41) on the nondominant side relative to Group B.

Furthermore, structural evaluations confirmed greater postural restoration in the integrated cohort. Group A achieved a greater reduction in forward head protrusion, evidenced by a between-group MD in OWD of -2.53 cm (95% CI: -2.78 to -2.28), reducing final postintervention values to near-normal alignment (1.12 ± 0.45 cm) compared with Group B (3.65 ± 0.82 cm). Correspondingly, the craniovertebral angle in Group A increased by a postintervention between-group MD of 6.26° (95% CI: 5.20-7.32) over Group B, reaching 47.82 ± 2.65° postintervention and approaching the recognized threshold for normal craniocervical alignment.

Mechanistically, these clinical variations are hypothesized to stem from reduced physical traction and compressive stress across the interscalene and costoclavicular intervals. This is clinically reflected in the marked postintervention reduction of neural tissue mechanosensitivity. Following the eight-week intervention, Group A achieved a higher rate of negative posttest conversions on the brachial plexus compression test, leaving only 10 individuals (18.2%) testing positive postintervention compared with 18 individuals (32.7%) in Group B.

Alleviating mechanical entrapment along the proximal nerve roots likely accounts for the downstream improvements observed in distal hand grip strength, as relieving C8-T1 lower trunk compression optimizes axoplasmic transport and motor unit recruitment to the intrinsic hand musculature. However, as direct real-time anatomical visualizations or electrodiagnostic verifications were not performed within this trial, these exact internal pathways remain speculative and warrant direct structural confirmation in future studies.

By shifting the therapeutic focus from localized symptom management to the proximal upper quarter kinetic chain, this study demonstrates that targeting cervicothoracic alignment directly addresses key contributing mechanisms of brachial plexus entrapment. The eight-week integrated postural training program offers physical therapists a structured, sequentially progressive protocol to restore muscle balance. The concurrent enhancement of distal hand grip strength alongside improvements in the craniovertebral angle and OWD indicates that proximal core and postural reeducation can directly enhance functional upper extremity capacity in individuals presenting with posture-related nTOS. Correcting these postural parameters provides clinical utility in restoring structural alignment and mitigating the risk of symptom recurrence.

These findings must be interpreted alongside key methodological limitations. The open-label nature of this physical intervention introduces potential performance or detection biases among investigators and participants. To mitigate this, data collection relied strictly on standardized, objective clinical metrics. A limitation of the present study is the lack of an a priori sample size calculation using standard power formulas (such as G*Power, Heinrich-Heine-Universität Düsseldorf, Düsseldorf, Germany) tailored specifically for independent MDs. Instead, the sample size was determined based on institutional feasibility, recruitment timelines, and precedents in regional comparative trials. While our final analyzed sample (n = 110) yielded robust statistical significance across all primary outcomes, future larger scale multicenter investigations should employ rigorous mathematical power modeling to further validate these effects. Additionally, outcomes were restricted to immediate posttreatment metrics following the eight-week intervention, meaning long-term retention data, durability, and recurrence patterns remain unquantified. Finally, structural screenings and evaluations relied completely on clinical special tests and photographic tracking rather than dynamic ultrasound, electromyography, or nerve conduction velocities to confirm real-time anatomical changes within the thoracic outlet space [20].

To expand upon the research trajectory of this study, several areas of future scope should be prioritized. First, long-term longitudinal studies incorporating multimonth or annual follow-up intervals are essential to track the long-term retention of postural corrections and determine rates of permanent symptom resolution. Second, integrating objective imaging modalities such as dynamic musculoskeletal ultrasound, magnetic resonance imaging, or electromyography would allow future trials to directly visualize structural cross-sectional changes within the interscalene triangle and retropectoralis minor spaces during specialized exercises. Finally, expanding into ergonomic and occupational subanalyses will allow researchers to explore the direct utility of this integrated protocol across specific high-risk, sedentary occupational cohorts (e.g., software engineers, dental hygienists), evaluating both its preventative and rehabilitative capacity in diverse real-world environments.

## Conclusions

An eight-week integrated postural training program demonstrated significantly greater improvements in pain, grip strength, postural alignment, and nerve tension in patients with nTOS compared with conventional exercises. While these findings highlight the clinical utility of targeting the proximal kinetic chain to resolve mechanical brachial plexus compression, this study's open-label design and immediate postintervention tracking must be noted. To establish the long-term durability, structural carry-over effects, and recurrence rates of these alignment corrections, further large-scale randomized controlled trials utilizing advanced diagnostic imaging and extended longitudinal follow-up periods are strongly recommended.
